# Harnessing Photon Density Wave Spectroscopy for the Inline Monitoring of up to 100 L Vinyl Acetate—Versa^®^ 10 Polymerization: Insights into Dispersion Dynamics and Mixing

**DOI:** 10.3390/polym17050629

**Published:** 2025-02-26

**Authors:** Stephanie Schlappa, Werner Pauer, Oliver Reich, Marvin Münzberg

**Affiliations:** 1 Institute for Technical and Macromolecular Chemistry, University of Hamburg, Bundesstraße 45, 20146 Hamburg, Germany; 2 Department of Physical Chemistry—InnoFSPEC, University of Potsdam, Am Mühlenberg 3, 14476 Potsdam, Germany; 3 Professorship of Knowledge and Technology Transfer, Faculty of Science, University of Potsdam, Am Mühlenberg 3, 14476 Potsdam, Germany

**Keywords:** photon density wave spectroscopy, emulsion polymerization, multiple light scattering, polyvinyl acetate, scale-up, inline monitoring

## Abstract

Photon Density Wave (PDW) spectroscopy is used as process analytical technology (PAT) in three batch sizes, 1 L, 10 L and 100 L, of polyvinyl acetate—neodecanoic acid vinyl ester (Versa^®^ 10) copolymerization. The effects on particle formation and growth are comparably analyzed. The data show comparability across scales up to a polymer volume fraction of around 0.15. Deviations beyond this suggest differences in particle growth dynamics. A detailed analysis of the dispersion dynamics and mixing properties provides an enhanced understanding compared to previous studies. Furthermore, the PDW spectroscopy data suggest inhomogeneity due to insufficient mixing at the beginning of the syntheses, despite very low feed-rates of the monomer mixture. PDW spectroscopy is thus capable of monitoring deviations in syntheses at different reaction volumes in real-time. These findings underline the potential of PDW spectroscopy not only for monitoring synthesis but also for enabling inhomogeneity analysis as a new application area. The integration of offline conversion and particle size measurements emphasizes the critical role of mixing efficiency in achieving optimal polymer dispersion properties and final product quality.

## 1. Introduction

Inline monitoring of industrially relevant manufacturing procedures, synthesis and automated processes has gained increasing relevance in recent years. The ongoing automation and implementation of smart sensor systems offers advantages for the producing industry. In polymerization synthesis, automated systems are widely used for automated dosing, stirring and other process control parameters. Smart solutions for inline monitoring, however, are rare and not generally used despite their potential economic impact [1,2,3]. The available inline control methods for polymer synthesis are not yet well optimized.

Vinyl acetate (VAc) and its polymers have been widely developed, used and intensively analyzed since the early days of polymerization theory [4,5]. But very little on inline analysis in liquid dispersions is published. Most analysis is conducted post-synthesis on dried films. Polyvinyl acetate (PVAc) blend films are formed and analyzed regarding swelling and mechanical characteristics [6,7,8,9]. For inline dispersion monitoring, a few methods, like in situ ATR-IR and inline Raman spectroscopy of the modification of the OH end groups by decrease of specific chemical vibrational bands and simultaneous conversion calculations, are possible [10,11,12]. The methods, however, can only be applied at low polymer concentrations and seem complicated and time consuming. Modeling of polymerization reactions is on a good path; however, it needs further optimization [13,14,15].

The inline monitoring of industrially relevant dispersions depends on process analytical technology (PAT). Problems with high-solid-content dispersions arise from the significant increase in emulsion viscosity with increasing solid content. The high amount of solids in the dispersion changes the macroscopic properties of the emulsion. Classical DLVO Theory is no longer applicable. The probability of coagulation is highly increased, leading to waste batches, increasing costs and safety issues [16,17,18,19]. To prevent coagulation and ensure a safe process, PAT tools, like Photon Density Wave (PDW) spectroscopy, can be implemented. Successful inline monitoring of high solid content (up to 63 wt % PVAc dispersions) by PDW spectroscopy of small reaction volumes up to 1 L has already been shown and reported [20,21,22].

In this research, PDW spectroscopy is further established as a powerful PAT tool that determines the absorption coefficient *µ*_a_ and reduced scattering coefficient *µ*_s_’ independently of each other without dilution or sampling. Jacob and Pauer reported on the scale-up process of the PVAc-Versa^®^ 10 synthesis [23]. The authors showed that inline particle size determination by PDW spectroscopy was successful. The authors also showed that inline measurements of the particle size agree very well with the theoretically calculated mean particle diameter. In contrast, measurements of particle sizes with dynamic light scattering (DLS) and disc centrifuge (DC) show huge deviations at solid contents over 30% polymer fraction [23].

Different approaches to synthesis scale-up are available but involve complicated steps to ensure a safe process [14]. The choice of polymerization technique has significant effects on the properties of the polymer product [24,25]. The upscaling of emulsion polymerization processes to large volumes might face many challenges. The nonlinear scalability of components often leads to safety issues, imperfect mixing and accumulation of reactive monomer [26,27,28,29]. Additional issues are reduced heat transfer [30], coagulation and reactor fouling by polymer precipitates and many more. The colloidal stability of the particles is affected in larger reaction volumes by dilution, reactor geometry and interactions between particles [31,32,33,34]. A proper scale-up procedure is important to maintain colloidal stability, prevent coagulum and ensure consistent material properties [35,36,37]. To guarantee safety and continuous product quality, PAT applications are highly valuable. However, only basic process analysis like flow rate control, pH control, temperature control and offline sample analysis are used in a standardized manner [34,38,39].

The main finding of this study is the ability to use PDW spectroscopy as a very early detection measure by means of the presented *I*_raw_ and *µ*_s_’ values in the initial reaction minutes. From these data, early deviations from an ideal process are detectable. These inconsistencies are proven by well-established methods like gravimetrical conversion control and particle sizing by DLS. The inconsistencies detected by PDW spectroscopy in *I*_raw_ are very useful to check if a polymerization process is progressing as desired. These findings are suitable to be transferred from the PVAc-copolymer analyzed here to various types of other polymer reactions and polymer systems.

The results and safety measures shown here for risk minimization of the given process are specific for the analyzed PVAc/Versa^®^ 10 copolymer synthesis. It is possible to adapt the results to other polymer reactions; safety measures, however, need to be revised and applied for each process individually.

## 2. Materials and Methods

The chemicals used for the starved-feed emulsion polymerization processes at different volume scales are described in Jacob and Pauer [23]. Table 1 shows the components used and their masses at the three different reaction volumes *V*_R_. Semi-continuous batch syntheses at a reaction temperature of *T*_R_ = 60 °C were conducted, as these showed better integration of Versa^®^ 10 into the copolymer and fewer safety issues with the generation of heat during the polymerization process [40,41]. To start a reaction, the amount of water was put into the reactor. The stirrer was placed at the height according to Table S1 in the Supplementary Materials. Stirring and N_2_ purging were started (cf. Table S2 in the Supplementary Materials). The reactors were equipped with automated temperature control by external thermostats (Julabo Cryo Compact F30-C thermostat, Huber Unistat Tango thermostat, Huber Unistat 405wl thermostat, regulated to constant jacket temperature by Huber, Offenburg, Germany). The reactor was heated to 60 °C. After a temperature equilibrium of about 10 min, polyvinyl alcohol Mowiol 4-88^®^ was added. The polyvinyl alcohol needed to be dissolved completely before starting the redox and monomer feed. This took several minutes, depending on the reactor size. The initial charge consisted consecutively of a 4.8% by mass polyvinyl alcohol aqueous solution.

**Figure 1:**
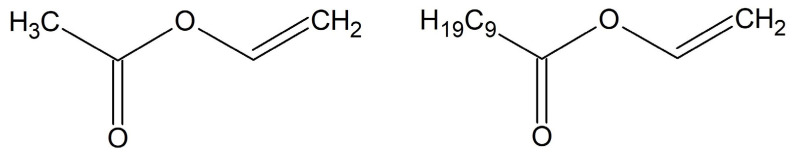
Representative chemical structures of monomers vinyl acetate (**left**) and neodecanoic acid vinyl ester (**right**).

The PVAc/Versa^®^ 10 copolymerization mechanism is explained in detail by Agirre et al. [40]. The chemical structures of the used monomers are shown in Figure 1. Versa^®^ 10 is less soluble in water than VAc; it diffuses faster into the active polymer particles and converts to polymer faster. As both monomers are water soluble to a certain extent, the diffusion into the active particles is fast [42]. The rate of polymerization can be assumed to be nearly equal to the feed-rate of the monomer. As polymerizations are run at a constant reaction temperature, it can be assumed that the rate of chain transfer to propagation stays constant throughout the experiment. Due to an excess of initiator radicals in the dispersion, termination reactions are suppressed to keep the polymerization alive and running [43].

PDW spectroscopy data analysis and evaluation were performed using LabView-based PDW spectroscopy software (v. 2025 Q1) developed, optimized and maintained by the University of Potsdam–innoFSPEC, Potsdam, Germany. The experimental data were checked for consistency. Data with a significant deviation were not used for further data treatment. At least three runs were conducted for each reaction volume to check the reproducibility of the experiments.

Input parameters like refractive index *n*, density *ρ* and particle volume fraction *ϕ* of the dispersion components were used equally, as in Jacob and Pauer, in order to keep the data comparable [23]. The refractive index of the pure polymer was determined by a customized refractometer DSR-λ by Schmidt and Haensch (Berlin, Germany); details can be found in our previous work [20]. The experimentally determined values are shown in Table 2. The formula used to determine the particle density is given below.

$$\rho_{Disp}=\frac{\rho_{D}\rho_{P}}{\omega \left[\rho_{D}-\rho_{P}\right]+\rho_{P}}↔\frac{1}{\rho_{Disp}}=\omega \left[\frac{1}{\rho_{P}}-\frac{1}{\rho_{D}}\right]+\frac{1}{\rho_{D}}$$

*ρ_Disp_*, *ρ_D_* and *ρ_P_* are the densities of the dispersion, the dispersant and the polymer. *ω* is the solid content of the dispersion. For the density of zero polymer content, the density of pure water was considered. These data are necessary for the experimental determination of the reduced scattering coefficient and absorption coefficient.

### 2.1. Photon Density Wave Spectroscopy

PDW spectroscopy has been optimized as a technique and established as a promising PAT tool. Detailed description of the technique, set-up [44,45] and application can be found in various publications [46,47,48,49,50]. As a PAT tool for the inline monitoring of polymerizations, PDW spectroscopy has been applied to small-scale reaction volumes in emulsion polymerization and other processes. It delivered reproducible results on the inline synthesis analysis, undiluted dispersion analysis and particle size analysis of high-solid-content, highly turbid liquid dispersions [20,22,46,51].

An advantage of inline PDW spectroscopy is its wide range of application. Using a simple optical fiber probe, it can be either implemented directly into the bulk reactor via an inlet port or it can be used in a bypass of the reactor where other PAT sensors are also implemented. Consequently, the volume, reactor set-up and probe positioning are key aspects. Measurements far away from the stirrer might not represent the conditions in the overall reactor. Sufficient mixing at the point of data acquisition is therefore crucial to produce a reliable PAT result.

A PDW spectroscopy measurement for one wavelength consists of a scan of 15 fiber distances *d*_f_ over a modulation frequency f, ranging from 10 to 510 MHz. Each distance *d*_f_ creates an intensity signal *I*_raw_ over the set frequency range. In a well-mixed dispersion, smaller distances result in higher intensity data, e.g., small *d*_f_ lead to higher *I*_raw_. Detected photons travelled a shorter measurement path length through the sample and have been scattered less often than at larger distances. For longer pathways, i.e., larger *d*_f_, lower intensities are expected as more scattering events and stronger absorption occur on the random walk of the photons through the sample from emission to the detection fiber. A detailed description of the measurement principle can be found in Bressel et al. [52]. Formula 27 in that paper describes the derivation of the optical coefficients *µ*_a_ and *µ*_s_’ from the distance-dependent measurements by PDW spectroscopy based on the theory of radiative transport [47,49].

As data are acquired based on manually set boundaries for the measurement distances *d*_f_ and used modulation frequencies, data curation after the measurement might be necessary. This is performed manually after the experiment has been completed. The initially set number of distances *d*_f_ and modulation frequencies *f* can be cut if the signal to noise ratio is too high. Too high noise might be interpreted as valuable data. This leads to errors in the analysis, as the used noise does not describe the particulate system. For each experiment, the data thereof are analyzed, and the used *d*_f_ and modulation frequencies are cut manually so that a proper fit of intensity and phase [52] can be applied by the algorithm in the used LabView analysis software (v. 2025 Q1).

### 2.2. Microwave Analyser

For offline conversion analysis, samples were dried with a Smart System 5 device from CEM (Kamp-Lintfort, Germany). A small sample of approx. 3 g was dried at 120 °C until mass consistency. Determination of the total solid content *ω* was performed by gravimetrical mass comparison between the wet and dried sample. This determined total mass (i.e., dried mass of polymer and stabilizing agent) was used for calculation of the respective volume fraction *φ*. The volume fractions have been calculated accordingly with the densities of the medium and polymer described in Table 2. The formula is shown below.

$$\varphi =\frac{\frac{\omega }{\rho_{P}}}{\frac{\omega }{\rho_{P}}+\left(\frac{1-\omega }{\rho_{D}}\right)}$$

with solid content *ω*; density of the polymer and the dispersant *ρ*_P_ and *ρ*_D_.

### 2.3. Dynamic Light Scattering

DLS measurements were performed on a Zetasizer Nano ZS from Malvern Instruments (Malvern Panalytical GmbH, Malvern, UK), using single-use polyethylene UV cuvettes with a diameter of 10 mm. The laser position and attenuator settings were determined automatically by the device itself. A scattering angle of 173° was used. The data analysis was based on the general-purpose model. For the sample preparation, one drop of dispersion sample was diluted in 5 mL demineralized water. The sizes shown here are shown as mean of three consecutive measurements, each individually consists of 18 measurements at 25 °C. The error bars indicate the deviation of the mean diameter between three measurements.

## 3. Results and Discussion

The results presented here are ordered in the way that the data would be temporally available during or after a synthesis performed in real-time in the laboratory. Therefore, inline-determined PDW spectroscopy data are shown for one synthesis first by means of the *I*_raw_ trends (Section 3.1), which are readily available in real-time while the synthesis proceeds. *I*_raw_ data are observed at the control unit of the PDW spectrometer. Further, inline *µ*_a_ and *µ*_s_’ data are simultaneously plotted and can be analyzed onsite. The data are analyzed for three reaction volumes (Section 3.2 and following). Next, offline data analysis of conversion by means of the solid content analysis and offline-determined particle size is additionally taken into account. Conversion measurements using a microwave analyzer are only available minutes after the time point of sampling due to sample withdrawal, preparation and sample analysis by drying at elevated temperatures. Data on the mean particle diameter by DLS are only available at a later point in time due to sampling, sample preparation by dilution, DLS analysis and subsequent evaluation.

In the end, in Section 3.5, the inline monitoring by PDW spectroscopy of the 100 L synthesis is shown to explore its suitability in high volume reactors and specify the effect of mixing on the dispersion properties.

### 3.1. Inline Process Analysis of PDW Spectroscopy Intensity Data

**Figure 2:**
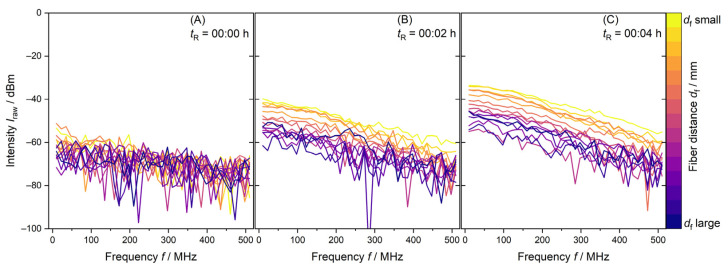
PDW spectroscopy *I*_raw_ data measurements at λ = 638 nm of the first minutes of a synthesis with 1 L reaction volume. (**A**) shows *I*_raw_ just before addition of monomer mixture, (**B**) after two minutes and (**C**) after four minutes of monomer dosing.

For rapid and high conversion and particle formation, a well-mixed and homogeneous reaction dispersion is crucial. Indications for an inhomogeneous dispersion can be observed in the PDW spectroscopy raw intensity data *I*_raw_. If a particle or droplet system, like an emulsion or dispersion, is homogeneously mixed and entities are evenly distributed, the *I*_raw_ signal shows a decrease from lowest to highest *d*_f_. An example is shown in Figure 2.

Figure 2 shows PDW spectroscopy *I*_raw_ data for a 1 L synthesis. *t*_R_ = 00:00 h shows a measurement of 15 fiber distances of the reaction mixture containing only water and redox initiator. Due to the absence of strongly scattering droplets and particles, measurement of this initial charge only leads to noise in the *I*_raw_ data in Figure 2A. *t*_R_ = 00:01 h indicates the addition of VAc/Versa^®^ 10 monomer mixture to the reaction vessel. Within two minutes and one PDW spectroscopy measurement, the *I*_raw_ values increase. An ordered decrease in *I*_raw_ from small to larger fiber distances *d*_f_ is established in Figure 2B. A further two minutes of reactant dosing causes *I*_raw_ to increase overall. A higher order of the fiber distances is visible in Figure 2C. These data are expected for a well-mixed, homogenous reaction mixture.

**Figure 3:**
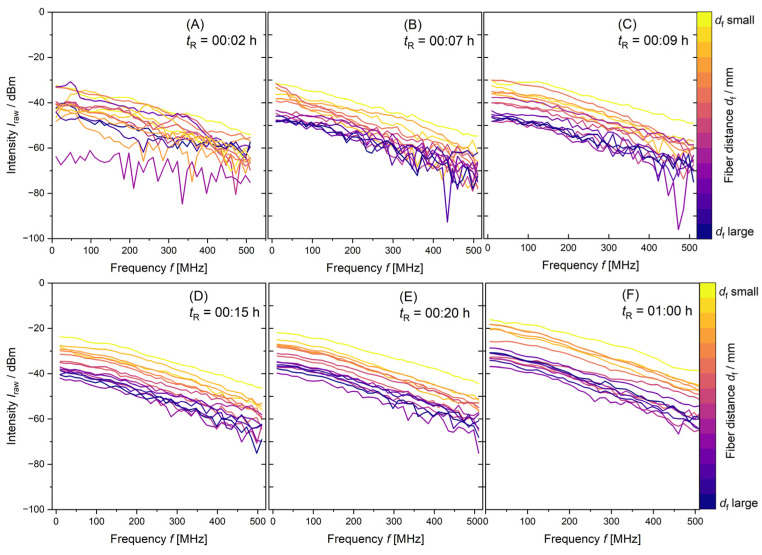
PDW spectroscopy *I*_raw_ data measurements at λ = 638 nm of a synthesis with 10 L reaction volume from *t*_R_ = 00:02 h to *t*_R_ = 01:00 h. With monomer mixture dosing time *t*_R_ increasing from (**A**–**F**) as shown and increasing order of *d*_f_.

Figure 3 shows PDW spectroscopy data *I*_raw_ over the modulation frequency *f* of all measured distances taken from a 10 L synthesis. From Figure 3A–F, the synthesis proceeds from *t*_R_ = 00:02 h to *t*_R_ = 01:00 h reaction time. The intensity *I*_raw_ values for the smallest *d*f (yellow line) and smallest modulation frequency *f* (10 MHz) increase with increasing reaction time from −30 to −18 dBm. The intensity of the measurement with highest *d*_f_ (purple line) and smallest modulation frequency increases from approx. −60 dBm with high noise to above −40 dBm. In a homogeneous dispersion, the measured intensity *I*_raw_ would decrease from *d*_f_ small to *d*_f_ large (from yellow to purple). With the proceeding polymerization, the reaction mixture becomes more turbid. More and larger particles contribute to absorption and scattering in the measurement path length. The detected inconsistencies by PDW spectroscopy in *I*_raw_ by means of a deviated order from the smallest to biggest measurement distances *d*_f_ are very useful to check if a polymerization process is progressing as desired.

With increasing reaction time *t*_R_, the order of the distance-dependent measurements from *d*_f, small_ to *d*_f, large_ of *I*_raw_ data changes. The irregularity of the measurements in Figure 3 for the 10 L synthesis proposes that inhomogeneity of the reaction mixture is present for reaction times up to at least *t*_R_ = 00:20 h. This might be caused by insufficient mixing. All dispersions analyzed in this paper have been produced with low feed-rates of the components. The monomer mixture is dosed into the dispersion at *r_Mon_*_1L_ = 1.2 mL min^−1^, *r_Mon_*_10L_ = 12 mL min^−1^ and *r_Mon_*_100L_ = 120 mL min^−1^, respectively. The stabilizer was completely added to the initial charge. With insufficient mixing, the high conversions needed for a starved feed polymerization are not guaranteed. The resulting particle properties will deviate from the desired product properties, leading to inconsistencies in product quality. As data acquisition is fast and the reaction mixture is not homogeneous, the measurements deviate strongly from each other. With increasing reaction time, particle formation and particle growth, the mixture becomes more homogeneous (see Figure 3D–F). PDW spectroscopy intensity measurements *I*_raw_ arrange to the measurement distance from smallest *d*_f_ to biggest *d*_f_, as anticipated.

The *I*_raw_ measurements for the 100 L synthesis also indicates incomplete mixing. The data can be found in Supplementary Material Figure S1.

### 3.2. Inline Analysis of Optical Coefficients—Initial Particle Nucleation and Growth

**Figure 4:**
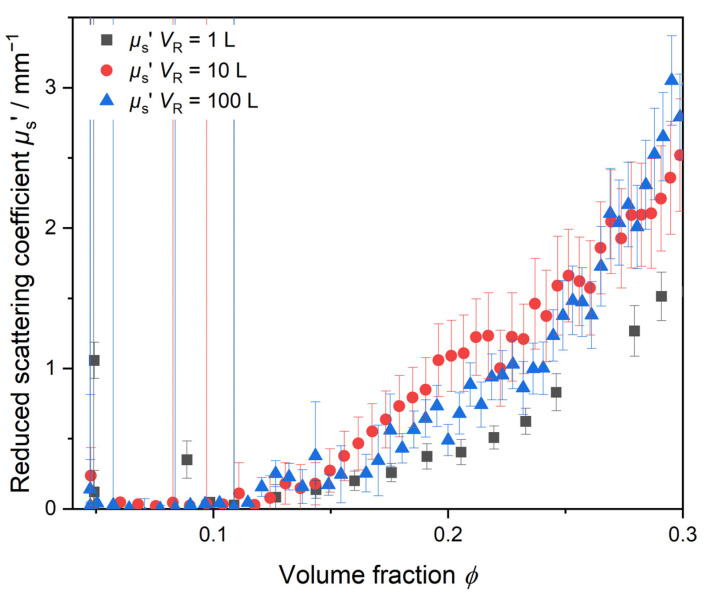
Initial polymerization phase monitored inline by PDW spectroscopy by means of the reduced scattering coefficient *µ*_s_’ inline trend at λ = 638 nm. Measurement of *µ*_s’_ at three different reaction volumes derived from a scale-up procedure from reaction volume of 1 L to 10 L and 100 L.

Additionally to the *I*_raw_ data, data on the reduced scattering coefficient *µ*_s_’ are measured inline by PDW spectroscopy and are readily available during the synthesis. Figure 4 shows the reduced scattering coefficient *µ*_s_’ measured inline in dependency of the particle volume fraction *ϕ* for batch sizes of 1 L, 10 L and 100 L. *ϕ* was calculated as the dosed mass of monomer and colloidal stabilizer at an assumed 100% conversion to polymer. The reaction temperature was kept constant at *T*_R_ = 60 °C. Until *ϕ* = 0.15, the scattering properties are comparable across all scales. The measured *µ*_s_’ indicates that up to *ϕ* = 0.15 particle nucleation and growth happens at a comparable rate of polymerization. Exceeding *ϕ* > 0.15, the measured *µ*_s_’ trends start to deviate from each other significantly.

Here, reliable PDW spectroscopy inline data can only be derived for volume fractions greater than *ϕ* > 0.1. Before, the scattering was too small to comply with the assumption of significant multiple scattering during the data analysis procedure. Due to the partial solubility of the monomer and small oligomers in water, larger, phase-separated polymer particles with a high refractive index in a significant amount, and thus strong scattering, are only formed later. The reduced scattering coefficient *µ*_s_’ shows a significant increase at all three reaction scales, starting around *ϕ* ≈ 0.11. The deviation of the *µ*_s_’ trends and different particle growth might be explained by the initial inhomogeneity of the respective reaction mixture.

From the *I*_raw_ data and the *µ*_s_’ trends, it can already be observed that the syntheses do not proceed in the same way, as anticipated. Deviations in the *µ*_s_’ trend clearly show that the dispersion properties are different after reaching a volume fraction of *ϕ* ≈ 0.15. PDW spectroscopy analysis is based on Mie theory. The *µ*_s_’ dependency on particle size according to Mie shows that smaller particles at low volume fractions lead to lower values for *µ*_s_’. As mixing of the 1 L sample is sufficient to create a homogeneous reaction mixture, many small active radical reaction centers are formed where the polymerization proceeds. Therefore, *µ*_s_’ is smallest for the 1 L sample up to *φ* < 0.3. The bigger reaction volumes show homogeneity issues, leading to less active reaction centers for polymerization to be formed and therefore larger *µ*_s_’ values.

### 3.3. Offline Dispersion Analysis

Further analysis of the synthesis was performed by offline conversion analysis by means of drying samples and determining the solid content and respective conversion.

**Figure 5:**
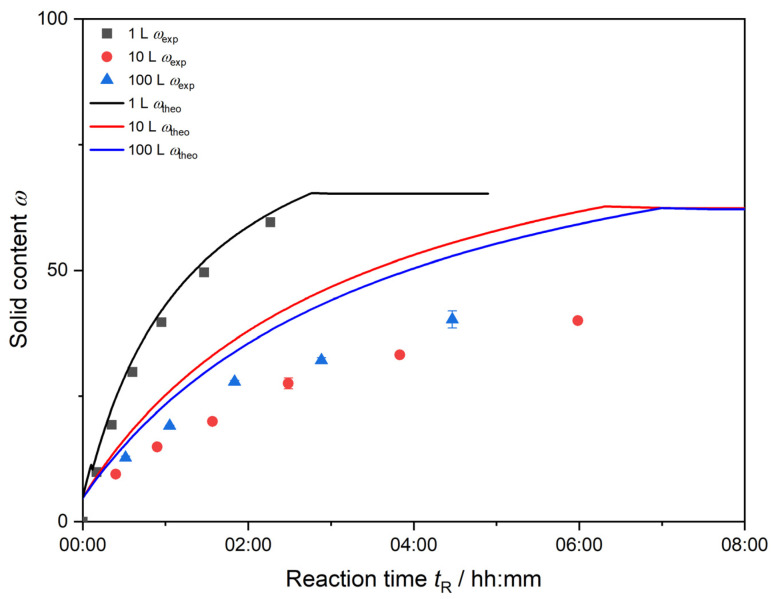
Calculation of theoretical solid content from 100% monomer conversion *ω*_theo_ (lines) and experimentally determined solid content *ω*_exp_ (symbols are color coded, respectively) of each reaction volume.

Figure 5 shows the experimental and theoretical solid content of each synthesis over the reaction time. The experimentally determined solid content *ω*_exp_ deviates from the theoretical amount of solids at 100% monomer conversion. This deviation is relatively small for a total reaction volume of 1 L. Due to sufficient mixing, the conversion of monomer to polymer is high, and little deviation between theoretical and experimental solid content *ω* is detected. The difference between *ω*_theo_ and *ω*_exp_ is the largest for the 10 L synthesis. The further each synthesis proceeds, the more divergence between theoretical solid content *ω*_theo_ and measured values *ω*_exp_ is observed. The redox initiator and monomer mixture are dosed into the dispersion at very low feed-rates. In the beginning of the synthesis, the small amount of monomer added to the dispersion is still quickly converted to a high extent. The further the synthesis proceeds, the deviation increases, and the non-converted monomer stays unreacted within the dispersion.

Note: For the 100 L synthesis, further measurements of the solid content and respective conversion calculation after *t*_R_ = 04:20 h were not possible due to heavy aggregation.

**Figure 6:**
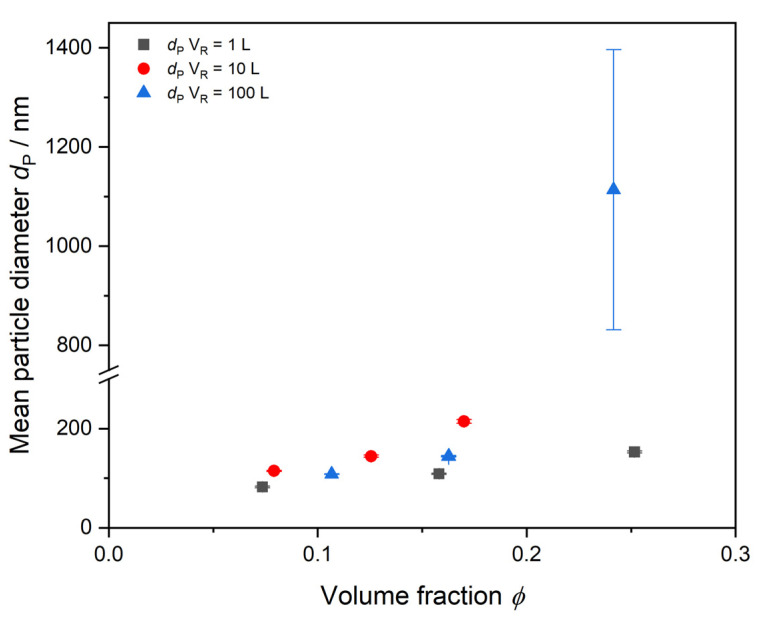
Volume-based mean particle diameter determined by DLS up to a volume fraction of *ϕ* = 0.3. Volume fractions have been calculated based on experimentally determined conversion data (cf. Figure 5). Depicted error values are standard deviations of three DLS replicate measurements.

Figure 6 shows offline-determined mean particle sizes by DLS for each reaction volume up to a volume fraction of *ϕ* = 0.3. The final products from the reactions should have the same properties like particle size and particle size distribution and therefore the same reduced scattering coefficient *µ*_s_’. From these offline measurements, it is evident that the growth of particles between the reaction volumes differs already at early reaction stages. Already at low volume fractions less than *ϕ* < 0.2, a difference in the determined mean particle size is detectable. The smallest reaction scale of 1 L reaction volume (black squares) produces the smallest particles. Exceeding *ϕ* > 0.2, the 100 L synthesis (blue triangles) contains particle aggregates in the micrometer size regime, with high deviations between the measurements indicated by the very big error bar. As the reduced scattering coefficient *µ*_s_’ strongly depends on the particle size within the dispersion, the deviations of the trends between the reaction scales are far earlier detected by PDW spectroscopy in real-time in Figure 3 and Figure 4. From the DLS data in Figure 6, it can be derived that the final particle sizes in the dispersions differ from each other. To achieve a successful scale-up procedure leading to equal properties across reaction scales, PDW spectroscopy can determine deviations already at low volume fractions in real-time much faster than common offline analysis.

### 3.4. Comparative Analysis Across Reaction Scales

**Figure 7:**
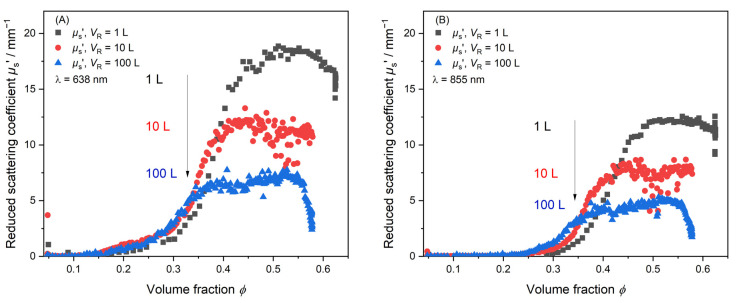
Inline monitoring of the synthesis process by PDW spectroscopy at *T*_R_ = 60 °C by means of *µ*_s_’. Measurement of *µ*_s’_ at three different reaction volumes. 1 L (black), 10 L (red) and 100 L (blue) at two different wavelengths. (**A**) *λ*_1_ = 638 nm and (**B**) *λ*_2_ = 855 nm measured simultaneously.

Figure 7 shows the reduced scattering coefficient *µ*_s_’ of three syntheses monitored over the complete reaction course in dependence of the volume fraction *ϕ*. *ϕ* > 0.57 is reached for all reaction volumes. Figure 7 shows PDW spectroscopy measurements at two different wavelengths, *λ*_1_ = 638 nm (Figure 7A) and *λ*_2_ = 855 nm (Figure 7B), respectively. An initial increase phase, a plateau phase and a decrease in scattering can be observed. A deviation of the *µ*_s_’ trends between different scales occurs around *ϕ* = 0.15 at λ = 638 nm and *ϕ* = 0.25 at λ = 855 nm. For all three reaction volumes, a plateau phase of *µ*_s_’ is found. In this plateau, the *µ*_s_’ value decreases with increasing reaction volume from *µ*_s_’_1 L_ = 18 mm^−1^ to *µ*_s_’_10 L_ = 12 mm^−1^ and *µ*_s_’_100 L_ = 7 mm^−1^. The difference in the plateaued *µ*_s_’ value indicates that the used scale-up procedure by Jacob et al. might not maintain particle properties in the final dispersion throughout reaction scales. It indicates that the particle growth phase between the reactions deviate from one another. To underline this, a second PDW spectroscopy measurement at *λ*_2_ = 855 nm is simultaneously analyzed. Figure 7B also shows a deviation in the final *µ*_s_’ values. Compared to Figure 7A, the discrepancy between the final *µ*_s_’ values in Figure 7B decreases. The final values are *µ*_s_’_1L_ ≈ 12 mm^−1^ to *µ*_s_’_10L_ ≈ 8 mm^−1^ and *µ*_s_’_100L_ ≈ 5 mm^−1^. Taking all the inline data produced by PDW spectroscopy into account, it is determined in real-time that the final particle properties will deviate from each other.

### 3.5. 100 L Synthesis Inline Analysis by PDW Spectroscopy—Effect of Mixing

In this section, the complete reaction of a 100 L synthesis is monitored by PDW spectroscopy. The reaction period of over *t*_R_ = 07:00 h is completely monitored. The PDW spectroscopy data shown are collected in real-time without any dilution of the dispersion. The PDW spectroscopy probe is directly inserted into the dispersion at all times of the synthesis.

**Figure 8:**
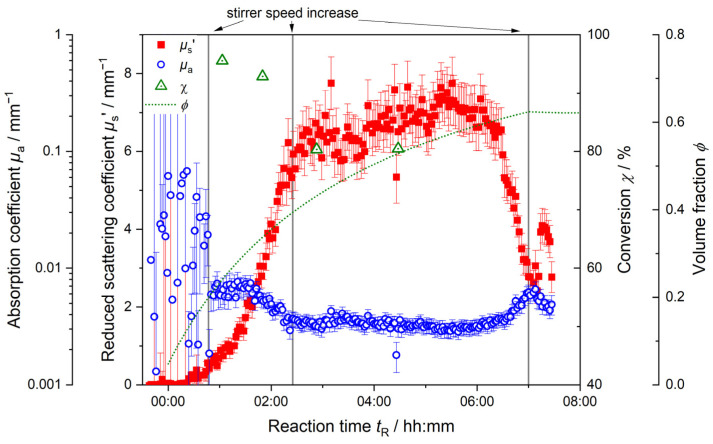
Inline monitoring of 100 L synthesis by PDW spectroscopy at *λ* = 638 nm with reduced scattering coefficient *µ*_s_’ (red) and absorption coefficient *µ*_a_ (blue). Offline-determined total conversion *χ* (green) and total volume fraction *ϕ* (green dotted line) are shown along the reaction progress. Vertical lines indicate manual increases of the stirrer speed.

Figure 8 displays the inline trends of the reduced scattering coefficient *µ*_s_’ and the absorption coefficient *µ*_a_ as well as the total volume fraction *ϕ* over time and the total conversion *χ* for a 100 L synthesis.

In the beginning of the reaction up until *t*_R_ = 00:50 h, the absorption coefficient *µ*_a_ (blue circles) does not yield consistent data points. Collected *µ*_a_ data are very noisy; error bars are high, and no trend is derivable. An increase in the stirrer speed at *t*_R_ = 00:50 h results in steady *µ*_a_ measurements. With the manual increase in stirrer speed, *µ*_a_ stays constant for approx. 00:30 h and decreases afterwards. This decrease in the total dispersion absorption is explained by the changes within the reaction dispersion. In the beginning up until the increase in stirrer speed, the system shown signs of inhomogeneity. The inhomogeneity itself is not at all increased. Due to this insufficient mixing, the assumed conditions for PDW spectroscopy measurements are not met, and PDW spectroscopy cannot deliver conclusive measurements for *µ*_a_. (PDW spectroscopy *I*_raw_ inline data are shown in Figure S1.) Further, with proceeding synthesis and increasing homogeneity due to the increased stirring speed after *t*_R_ = 00:50 h, the absorption decreases. This decrease is caused by the combined changes happening in the dispersion. As absorption is a sum parameter, it is influenced by all the entities available in the dispersion. In the beginning of the synthesis, the absorption of the dispersion is mostly influenced by the high absorption of the aqueous medium und a high number of active radicals. It was found before that created radicals from the initiator and monomers show a high absorption [53]. Due to insufficient mixing, possible monomer droplets in the dispersion also contribute to the higher *µ*_a_ values. With proceeding synthesis, the radical number and residual monomer in the dispersion decreases. Simultaneously, polymer particles start to form and grow. As the absorption of polymer particles is lower than that of the radicals and the aqueous medium, the overall absorption of the dispersion decreases. After a second manual stirrer increase at *t*_R_ = 02:30 h, it levels off at values around 0.003 mm^−1^.

Only after approx. 06:00 h of reaction time, the absorption starts to increase again without any applied extrinsic changes. This increase is caused by the aggregation of polymer particles in the dispersion. The total number of particles decreases as individual particles coagulate and form aggregates. A further stirrer speed increase was applied at *t*_R_ = 07:00 h in order to counteract coagulation of the dispersion. However, coagulation could not be averted. The final product achieved was a paste rather than a liquid dispersion.

In Figure 8, the trend of the reduced scattering coefficient *µ*_s_’ (red squares) starts to increase slowly in the beginning at *t*_R_ = 00:15 h. The formation of particles starts to influence the scattering properties of the dispersion. The slope of *µ*_s_’ increases continuously as polymer particles are formed and continue growing. The manual increase in stirrer speed at *t*_R_ = 00:50 h does not show such a significant effect on *µ*_s_’ as it has on *µ*_a_. *µ*_s_’ continues to grow; its slope becoming steeper as more polymer particles as scattering entities are formed and increase in size, contributing to the overall scattering. At the second manual increase in stirring speed at *t*_R_ = 02:30 h, *µ*_s_’ still increases for a while, but the acquired datapoints become noisier, and a slight overall increase is observed up until approx. *t*_R_ = 06:00 h. During the course of the reaction, the changes in scattering become less pronounced; initially, *µ*_s_’ increases strongly with a steep slope. With the continuous addition of monomer to the dispersion (green dotted line), the particles continue to grow. However, the changes in their volume, the overall volume fraction and particle number are less pronounced, creating a decreasing slope of *µ*_s_’. After *t*_R_ = 06:00 h, *µ*_s_’ suddenly decreases significantly. Both optical coefficients *µ*_a_ and *µ*_s_’ change. As discussed before here and in Schlappa et al., this can be a sign of particle aggregation in the dispersion [20].

In Figure 8, for the 100 L synthesis, the conversion χ (green triangles) decreases from over 95% to approx. 80%. Due to the initial inhomogeneity (cf. Figure 4, Figure 5 and Figure 7), a conversion of only 96% is achieved in the beginning. Although very low feed-rates of monomer are used, it is not fully converted. The conversion decreases over the whole course of the reaction. Despite manual increases in stirring speed, the initial lack of full conversion cannot be retrieved. As the monomer is continuously fed into the dispersion, it is not completely converted. The running reaction cannot recover the effects the insufficient mixing had on the conversion. The presumed starved-feed process is not reached. Conversion measurements after *t*_R_ = 04:20 h were not possible here. The high solid contents of the samples drawn from the reactor lead to inconclusive microwave analyzer results. The inline monitoring of the synthesis shows that the initial inhomogeneities detected in the PDW spectroscopy *I*_raw_ measurements are also detectable in the noisy inline data acquisition of *µ*_a_ and the low conversion of the reaction.

## 4. Conclusions

For the first time, the suitability of monitoring 10 L and 100 L reaction volumes with inline PDW spectroscopy for high-solid-content emulsion polymerization synthesis has been described in detail. It is shown that the technique delivers inline data on the optical coefficients without dilution of the dispersion. The feasibility of PDW spectroscopy for the 100 L reaction scale with industrially relevant dispersion properties was successfully demonstrated. The PVAc copolymerization was successfully monitored in real-time. It was shown here that PDW spectroscopy data provide much physical and chemical information about polymerization processes. These findings can be adapted for various other emulsion polymerization systems. On top of the optical coefficients of a polymer dispersion, insights into its dynamics can be drawn. For the scale-up process adapted here with reaction volumes from 1 L to 10 L and 100 L, the reduced scattering coefficient *µ*_s_’ increases comparably only for up to *ϕ* = 0.15, indicating similar initial particle nucleation and growth rates throughout the different reaction volumes. However, as the reactions proceed, deviations in both measured inline trends of *µ*_a_ and *µ*_s_’ suggest differences in the final dispersion properties. It is shown by DLS particle size analysis that the particle size indeed starts to grow deviant to that in the Model 1 L system. These data are particularly valuable and relatable to various polymerization processes as they capture real-time changes in optical properties, which reflect critical aspects of the reaction’s progression in particle growth. Initial deviations between the syntheses can already be seen inline via PDW spectroscopy *I*_raw_ data in real-time in the first synthesis minutes.

Instead of relying on experience, data-driven knowledge can be used to ensure safe operation of large-scale syntheses and consistent product quality. PDW spectroscopy inline data reveal these early reaction inconsistencies. Insufficient mixing and resulting inhomogeneity, especially in the larger volumes, hint that starved-feed conditions are not achieved. It is detected that the scale-up procedure is not feasible to deliver identical dispersion properties. This was only found after advanced analysis of PDW spectroscopy data in combination with offline conversion data. Earlier studies based on basic data evaluation could not deliver these progressive results. The inline data highlight the importance of sufficient mixing at early reaction stages to achieve high conversion values. Further, the implemented PDW spectroscopy as PAT was able to indicate coagulation incidents during synthesis. Both inline trends *µ*_a_ and *µ*_s_’ significantly change during coagulation, and countermeasures could be introduced to the dispersion to prevent it.

## Figures and Tables

**Table 1:** Experimental set up of semi continuous emulsion polymerization reactions. Initial masses of water and polyvinyl alcohol Mowiol 4-88^®^ in the initial charge (iC) in the respective reactor as well as total mass of monomer mixture VAc—neodecanoic adic Versa ^®^ 10 dosed during the reaction with the given feed-rate.

Reaction Volume *V*_R_	Water_iC_ /g	Stabilizing Agent Mowiol 4-88^®^_iC_ /g	Monomer Mixture 9:1 VAc:Versa^®^ 10 Total /g	Feed-Rate Monomer Mixture /g min^−1^	Initiator Redox Pair Ascorbic Acid (3.4% _aq_) TBHP (3.4% _aq_) /g	Feed-Rate Redox Initiators /mL h^−1^
1 L	385	19.7	819	1.2	31.9	10
10 L	2442	123	4543.4	12	374.2	25
100 L	27,503	1383.4	60,017	120	4013.4	530.4

**Table 2:** Experimentally determined refractive index *n* and density *ρ* data used in this research.

Component	Density *ρ* at *T* = 20 °C/gcm^−3^	Refractive Index *n* at *λ* = 638 nm at *T* = 20 °C	Refractive Index *n* at *λ* = 855 nm at *T* = 20 °C
Dispersion Medium	0.997	1.3315	1.3272
PVAc/Versa^®^ 10 copolymer particle	1.214	1.4849	1.4769

## Data Availability

The original contributions presented in the study are included in the article/supplementary material, further inquiries can be directed to the corresponding author.

## Supplementary Materials

The following supporting information can be downloaded at: https://www.mdpi.com/article/10.3390/polym17050629/s1, Figure S1: PDW spectroscopy Iraw data measurements at λ = 638 nm of a synthesis with 100 L reaction volume from tR = 00:02 h to tR = 00:38 h. Initial measurements up to at least tR = 00:22 h show irregularities in the order of Iraw and df. This might be caused by an inhomogeneous dispersion due to insufficient mixing; Figure S2: Inline monitoring of 10 L synthesis by PDW spectroscopy at λ = 638 nm with reduced scattering coefficient *µ*_s_’ (red) and absorption coefficient *µ*_a_ (blue). Offline determined total conversion Χ (green) and total volume fraction *ϕ* (green dotted line) are shown along the reaction progress. Vertical lines indicate manual increases of the stirrer speed; Table S1: Overview of the measurements of the experimental set-up of all three reactor sizes at different polymerization reaction volumes V_R_.with H as height of the reactor, D as diameter of the reactor, d as diameter of the used anchor stirrer, H1 as height of initial Charge before starting the reaction and h as height from the bottom of the reactor to the anchor stirrer; Table S2: Initial stirring rates for the different reaction volumes V_R_.
