# Supplementary material for: Harnessing Photon Density Wave Spectroscopy for the Inline Monitoring of up to 100 L Vinyl Acetate—Versa® 10 Polymerization: Insights into Dispersion Dynamics and Mixing
Source: Polymers (Basel). 2025 Feb 26;17(5):629. doi: 10.3390/polym17050629 (PMC11902584; doi:10.3390/polym17050629)
Supplement: Supplementary file 1 [file polymers-17-00629-s001.zip › polymers-3466163-supplementary.pdf]

Supplementary material

Table S1. Overview of the measurements of the experimental set-up of all three reactor sizes at different polymerization reaction volumes V_R, with H as height of the reactor, D as diameter of the reactor, d as diameter of the used anchor stirrer, H1 as height of initial charge before starting the reaction and h as height from the bottom of the reactor to the anchor stirrer.

Reactor dimensions (mm)	V_R = 1 L	V_R = 10 L	V_R = 100 L
H	205	270	600
D	120	140	480
d	104	208	416
H1	30	60	120
h	10	15	50

Table S2. Initial stirring rates for the different reaction volumes V_R.

Reactor volume V_R	Initial stirring rate /rpm
1 L	300
10 L	75
100 L	60

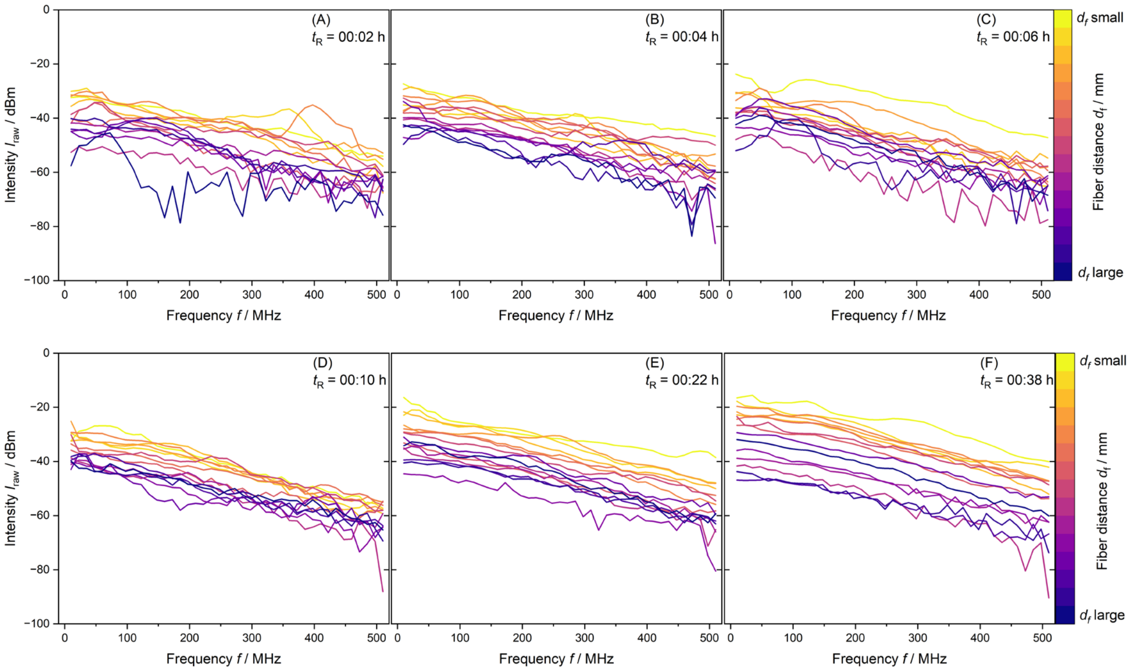

Figure S1. PDW spectroscopy I_{raw} data measurements at λ = 638 nm of a synthesis with 100 L reaction volume from (A) at t_R = 00:02 h to (F) at t_R = 00:38 h. Initial measurements up to at least (E) t_R = 00:22 h show irregularities in the order of I_{raw} and d_f. This might be caused by an inhomogeneous dispersion due to insufficient mixing.

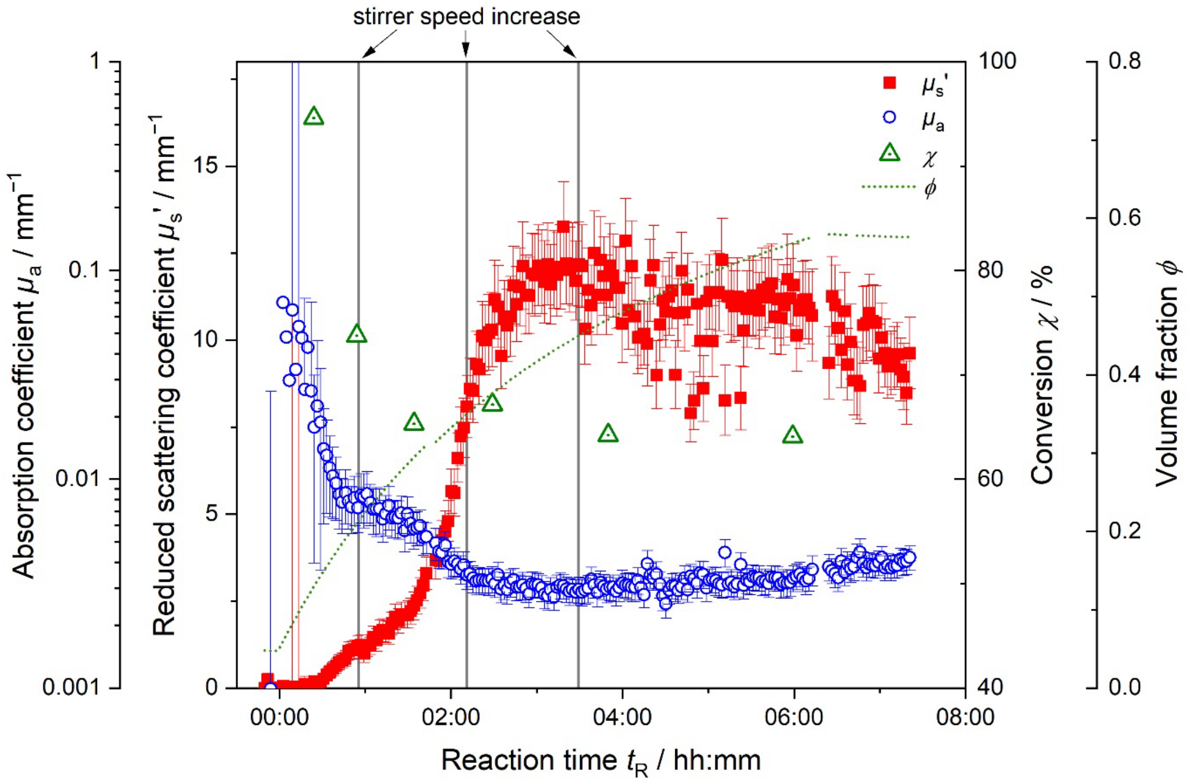

Figure S2. Inline monitoring of 10 L synthesis by PDW spectroscopy at λ = 638 nm with reduced scattering coefficient μ_s' (red) and absorption coefficient μ_a (blue). Offline determined total conversion χ (green) and total volume fraction ϕ (green dotted line) are shown along the reaction progress. Vertical lines indicate manual increases of the stirrer speed.

S2 displays the reduced scattering coefficient μ_s', the absorption coefficient μ_a, the total volume fraction ϕ and the total conversion χ over time for a 10 L synthesis. In the beginning the absorption of the dispersion lies within the order of 0.02 – 0.06 mm^{-1}. These high values, compared to the expected absorption of water and polymer might arise due to the high absorption of the generated radicals. With proceeding synthesis, the absorption decreases more than one order of magnitude. As the synthesis proceeds particles are formed and the number of radicals decrease, leading to a lower overall absorption. The formed particles outnumber the radicals and exhibit a lower absorption. With proceeding particle formation and particle growth, the contribution of the radical absorption decreases rapidly and lowers the total μ_a to values around 0.003 mm^{-1}.

For the 10 L synthesis an increase in the stirrer speed by means of revolutions per minute (*rpm)* at t_R = 0:57 h results in a lower slope of μ_a and smaller error bars. In the initial phase of the reaction the addition of monomer induces a more pronounced change to the dispersion compared to later stages of the reaction. Continuous monomer addition at a constant feed-rate results in a decreasing relative monomer fraction over time. While the total amount of monomer increases, the relative change in monomer content decreases substantially. Insufficient mixing as proposed by inline PDW spectroscopy I_{raw} data in Figure 3 in the manuscript prevents full monomer conversion. Initially with excess of initiator and a high concentration of active radicals, conversion remains high, χ > 90 %.

In Supplement Figure S 2 μ_s' increases after the addition of monomer mixture at t_R = 00:15 h. The first increase in stirrer speed at t_R = 00:57 h inflicts a steeper slope of μ_s'. With increasing the stirrer speed, better mixing and homogeneity of the dispersion is approached which promotes particle growth and increases the overall scattering.

A second increase in stirrer speed at t_R = 02:10 h causes μ_a to level off. At the same time a slight increase in the conversion χ happens. μ_s' still rises up to a third stirrer speed

increase at t_R = 03:30 h plateauing at around μ_s' = 11 mm^{-1}. A final conversion of merely $\chi \geq 65$ % is reached. Due to the inhomogeneity of the dispersion in the beginning, high conversions of monomer to polymer cannot be recovered. This shows the extreme necessity for an inline control of the particle dispersion and homogeneity from the early beginning on.

The changes of the two trends of μ_a and μ_s' towards the end of the synthesis might already be an indication for coagulation of the particles. This was however not further analyzed here.
